# Effect of the Combination of Whole-Body Neuromuscular Electrical Stimulation and Voluntary Exercise on Metabolic Responses in Human

**DOI:** 10.3389/fphys.2019.00291

**Published:** 2019-03-20

**Authors:** Kohei Watanabe, Takahiro Yoshida, Tomoki Ishikawa, Shuhei Kawade, Toshio Moritani

**Affiliations:** ^1^Laboratory of Neuromuscular Biomechanics, School of International Liberal Studies, Chukyo University, Nagoya, Japan; ^2^MTG Co., Ltd., Nagoya, Japan; ^3^School of Social Science Health and Sport Sciences, Chukyo University, Toyota, Japan; ^4^Faculty of Sociology, Kyoto Sangyo University, Kyoto, Japan

**Keywords:** electrical muscle stimulation, electrical myostimulation, sarcopenia, life-style-related diseases, lactate, high-intensity interval training

## Abstract

**Purpose:**

Since neuromuscular electrical stimulation (NMES) can recruit high-threshold motor units and enhance glucose metabolism, the combination of NMES and voluntary low-intensity exercise would induce both anerobic and aerobic energy consumptions and this type of exercise could be more efficient and effective than conventional exercise regimens. We aimed to investigate metabolic responses and muscle fatigue during whole body NMES (WB-NMES), voluntary exercise, and their combination.

**Methods:**

The blood lactate concentration and maximal voluntary contraction were measured before and after specified exercises: WB-NMES (E), voluntary exercise (V), and their combination (VE), and expired gas was sampled during the exercises in thirteen healthy young men. Each exercise was conducted for 15 min and interval between exercise was > 48h.

**Results:**

Energy expenditure and $\dot{\text{V}}{\text{O}}_{2}$ relative to the body mass during VE were significantly higher than during V and E (*p* < 0.05). The Respiratory gas exchange ratio (RER) during both E and VE was higher than during V (*p* < 0.05), and the blood lactate concentration after VE was significantly higher than after V and E (*p* < 0.05). Although $\dot{\text{V}}{\text{O}}_{2}$ relative to the body mass was 18.6 ± 3.1 ml/min/kg and the metabolic equivalent was 5.31 ± 0.89 Mets, the blood lactate concentration reached 7.5 ± 2.7 mmol/L after VE.

**Conclusion:**

These results suggest that the combination of WB-NMES and voluntary exercise can enhance the metabolic response to a level equivalent to high intensity exercise under the net physiological burden of low-middle intensity exercises.

## Introduction

It is well known that exercise is essential for the prevention and management of metabolic diseases, such as type 2 diabetes mellitus (Sigal et al., 2006). Aerobic exercise for conditioning metabolic and cardiovascular systems and resistance exercises for strengthening skeletal muscles are major component of the exercise regimen recommended for such patients. Also, since high-intensity exercise like resistance training can enhance glucose metabolism, this type of exercise should be applied for not only the prevention and management of metabolic diseases but also for improvements in muscle mass or strength (Colberg et al., 2010). However, resistance training may lead to high-impact stress on joints and ligaments or orthopedic disorders.

Neuromuscular electrical stimulation (NMES) could be a useful alternative method of resistance training for people unable to perform high intensity exercise (Lake, 1992; Banerjee et al., 2005; Hortobagyi and Maffiuletti, 2011). It has been shown that the motor unit recruitment pattern during NMES is random and does not follow the size principle, NMES can activate motor units or muscle fibers with a high recruitment threshold even during low intensity electrical stimulation (Gregory and Bickel, 2005; Jubeau et al., 2007; Bickel et al., 2011). This physiological response can enhance glucose metabolism (Hamada et al., 2003, 2004a) and/or muscle hypertrophy (Hasegawa et al., 2011). Recently, the combined regimen of whole-body NMES (WB-NMES) and voluntary exercise was developed (Kemmler et al., 2012, 2014), since this type of regimen is both time-saving and orthopedically gentle. In fact, positive effects of WB-NMES on the muscle mass and function and cardio-metabolic risk factors have been reported (Kemmler and von Stengel, 2013; Wittmann et al., 2016). However, physiological responses during WB-NMES have not been fully clarified. While some studies measured physiological responses such as cardiovascular and/or metabolic responses during NMES application to local muscles (Hamada et al., 2003, 2004a), few studies have quantified physiological responses during WB-NMES. For example, Kemmler et al. (2012) showed energy expenditure during low-intensity resistance training with WB-NMES of 17% higher than that without WB-NMES (Kemmler et al., 2012). Although this previous study quantified energy consumption during the combination of voluntary exercise and WB-NMES, the detailed metabolic response was not investigated to clarify the physiological implication of combining the two types of exercises.

The purpose of the present study was to investigate metabolic responses and muscle fatigue during WB-NMES, voluntary exercise, and their combination. When NMES is applied to pairs of agonist-antagonist muscles on voluntary contraction, it should induce eccentric contraction of antagonist muscles and co-contraction between agonist muscles and NMES-elicited contraction of antagonist muscles (Lepley et al., 2015). This could increase physiological burdens on the muscles and energy expenditure. We thus hypothesized that 1) energy expenditure during the combination of voluntary exercise and WB-NMES is greater than during WB-NMES, voluntary exercise, and their summation, 2) muscle fatigue after the combination of voluntary exercise and WB-NMES is greater than those of WB-NMES and voluntary exercise, and 3) the addition of WB-NMES to voluntary exercises enhances glucose metabolism. These hypotheses were tested by comparing the energy expenditure, oxygen consumption, blood lactate concentration, and maximal voluntary contraction (MVC) during and after WB-NMES, voluntary exercise, and their combination, respectively. We set hypothesis 1 as the primary one to answer the main question of whether the combination of WB-NMES and voluntary exercise induces greater energy consumption.

## Materials and Methods

### Subjects

Thirteen healthy young men (age: 20.7 ± 0.9 years, height: 172.5 ± 5.5 cm, body mass: 63.1 ± 7.6 kg) volunteered for the present study. They did not participate in regular endurance/strength training or competitive athletic events. All subjects gave written informed consent for the study after receiving a detailed explanation of the purposes, potential benefits, and risks associated with participation. They were healthy with no history of any musculoskeletal or neurological disorders. All study procedures were conducted in accordance with the Declaration of Helsinki and research code of ethics of Chukyo University, and were approved by the Committee for Human Experimentation of Chukyo University (2017-002 and -057).

### Study Design

**FIGURE 1:**
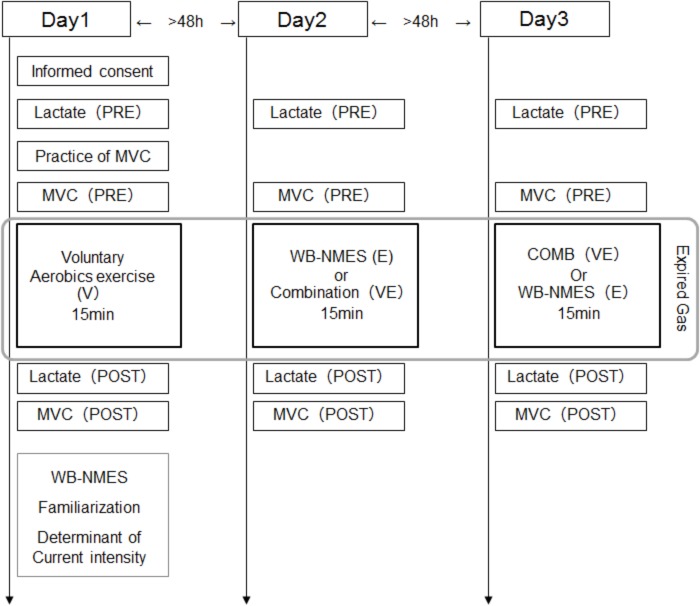
Experimental protocols in this study.

Subjects came to the laboratory three times, separated by at least 48 h intervals (2 of 26 cases were separated by only 24 h). On all three days, the blood lactate concentration and MVC were measured before and after a given exercise, i.e., WB-NMES (E), voluntary aerobic exercise (V), or their combination (VE), and expired gas was sampled during a given exercise. On the first day, subjects performed V and familiarization with and determination of the stimulation intensity in WB-NMES were conducted after its completion (Figure 1). On the second or third days, E or VE were randomly applied. Each exercise program was conducted for 15 min (Figure 1).

### Measurements

We measured expired gas from mask covering the mouth and nose during exercise using the breath-by-breath method (AE310S, Minato Medical Science Co., Ltd., Osaka, Japan). $\dot{\text{V}}{\text{O}}_{2}$ relative to the body mass and the respiratory gas exchange ratio (RER) were calculated from sampled gas. The energy expenditure and metabolic equivalent were also calculated from $\dot{\text{V}}{\text{O}}_{2}$ and the body weight of each subject for each exercise. To test hypothesis 1, summation of $\dot{\text{V}}{\text{O}}_{2}$ relative to the body mass for V and E exercises was performed. The basal metabolism (3.5 ml/min/kg) for an exercise was subtracted from the summed V and E in $\dot{\text{V}}{\text{O}}_{2}$ relative to the body weight for comparison with that in VE.

The blood lactate concentration was measured just after exercise with the lactate oxidase method using an automated analyzer (Lactate Pro; Arkray, Kyoto, Japan) and 5 μL of blood obtained from the fingertip before and after exercise (Watanabe et al., 2014). Subjects sat in a chair during blood sampling. The mean value of two samples for one measurement was used for further analysis.

Maximal voluntary contraction during isometric knee extension and elbow flexion was measured before and after exercise to assess muscle fatigue of the knee extensor (quadriceps femoris) and elbow flexor (biceps brachii) muscles. The subjects were asked to gradually increase their isometric contraction force from the baseline to maximum in 2-3 s and then sustain it maximally for 2 s. Two MVCs were performed before the given exercise and the highest MVC value was used for analysis. After the specified exercise, the subjects performed one MVC. For knee extension, the subjects sat in a custom-made dynamometer and their ankle was fixed to the force transducer with a 90° knee joint angle and a 120° hip joint angles (Watanabe, 2018). For elbow flexion, the subjects sat in a chair and their forearm and wrist were fixed to the force transducer with a 120° elbow joint angle (Watanabe et al., 2015).

### Voluntary Exercise

The subjects performed voluntary exercise like body weight resistance training for the whole body during V and VE. This exercise program includes 60 s of stepping as warm-up, 96 s of horizontal squat with pec deck fly (16 reps), 180 s of pec deck fly (30 reps), 198 s of lunge with twist (32 reps), 96 s of horizontal squat with arm curl (16 reps), 5 sets of knee-to-elbow as much as possible in 5 s with 10 s of inter-rest, and resting phases between these exercises (Total: 15 min.). During V and VE, the experimenters showed the subject the specified exercise and instructed them on it.

### WB-NMES

**FIGURE 2:**
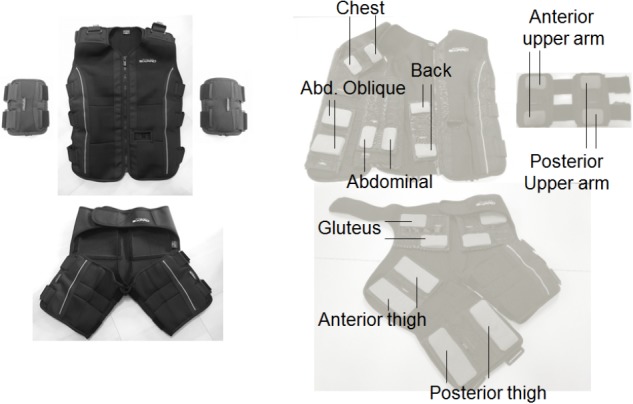
Arm band, vest, and shorts for whole-body neuromuscular electrical stimulation (Left panel) and their inside electrode locations for each muscle group (Right panel).

NMES was applied to the anterior and posterior upper arm, chest, back, abdominal, abdominal oblique, gluteus and anterior and posterior thigh muscles using the custom-made stimulator based on a commercially developed NMES device (SIXPAD, MTG Ltd., Nagoya, Japan) (Watanabe et al., 2017). Silicon-rubber electrodes covered by wet clothes were used as stimulation electrodes. Pairs of electrodes were placed on the individual muscle groups, with the details of each electrode shown in Table 1. These electrodes were set on the inside of an arm band, vest, and shorts that can be tightened by adjustor belts (Figure 2) and were connected to small wireless stimulators (48.4 x 36.5 x 15.0 mm, 27 g) that are synchronized among stimulators by a control unit. The fundamental duty cycle of NMES was a 4 s stimulation with a 4 s pause and the stimulation frequency was 20 Hz (Moritani et al., 1985). We also applied 2 Hz stimulation during the warm-up and resting phases. Biphasic square current pulses with a 100 μs duration were applied. The maximal electrical potential and current intensity of this device were 50 V and 4.85 mA, respectively. The current intensity was determined as the highest intensity that the subject could perform voluntary exercise without discomfort in each muscle group. The subjects chose 20∼80% of the maximal current intensity of the device. Familiarization with and determination of the stimulation intensity for each subject for WB-NMES were performed after measurements of V on Day1 (Figure 1). During VE and E, the current intensity for each muscle group was dynamically changed between 70-100% of the maximal tolerant current intensity for each subject following joint movement in voluntary exercise. For example, current intensities for the chest, back, gluteus, and posterior thigh muscle groups were 100% of the maximal tolerant current intensity for each subject and those for arms, abdominal, and abdominal oblique muscles were 70% of the maximal tolerant current intensity for each subject during the horizontal squat with pec deck fly (E1, Table 2).

**Table 1:** Electrode size and inter-electrode distance for each muscle group.

	Electrode size (mm)	Inter-electrode distance (mm)
Anterior upper arm	37 × 63	71
Posterior upper arm	37 × 63	71
Chest	37 × 63	53
Back	37 × 63	125
Abdominal	52 × 90	50
Abdominal oblique	37 × 63	40
Gluteus	52 × 90	51
Anterior thigh	51 × 144	102
Posterior thigh	51 × 144	64


**Table 2:** Stimulation frequency and intensity during whole body neuromuscular electrical stimulation.

	W-up	E1	E2	E3	E4	E5	Rest
**Frequency (Hz)**					
	2	20	20	20	20	20	2
**Stimulation intensity (% of maximum for each subject)**					
Anterior upper arm	100	70	0	50	100	100	30∼100
Posterior upper arm	100	70	0	50	100	100	30∼100
Chest	100	100	100	70	70	100	30∼100
Back	100	100	100	70	70	100	30∼100
Abdominal	100	70	100	100	70	100	30∼100
Abdominal oblique	100	70	70	100	70	100	30∼100
Gluteus	100	100	70	100	100	100	30∼100
Anterior thigh	100	100	70	100	100	100	30∼100
Posterior thigh	100	100	70	100	100	100	30∼100


*W-up, stepping as warm-up; E1: horizontal squat with pec deck fly; E2, Pec deck fly (30 reps), E3, Lunge with twist; E4, Horizontal squat with arm curl; E5, Knee-to-elbow.*

During VE, contraction and relaxation times of NMES were synchronized with voluntary exercise, i.e., NMES was applied during muscle lengthening or shortening by joint movements. For E, the same WB-NMES program with VE was applied while the subjects were in a supine position on a bed without performing any voluntary contractions.

### Statistics

All data are presented as the mean and standard deviation. To test the effect of the exercise type, one-way ANOVA was applied to $\dot{\text{V}}{\text{O}}_{2}$ relative to the body mass, RER, blood lactate concentration after exercises, and MVC after exercise relative to that before exercise. When there was a significant effect of the exercise type based on ANOVA, the parameters among V, E, and VE were compared by *post hoc* tests such as Tukey HSD. We also compared $\dot{\text{V}}{\text{O}}_{2}$ relative to the body mass between the summation of V and E that were separately performed and VE using the paired *t*-test. The level of significance was set at 0.05. The epsilon-squared estimate of effect size (𝜀^2^) was additionally calculated and this value from 0 to 1 indicates no relationship to a perfect relationship (Tomczak and Tomczak, 2014). Statistical power was calculated in *post hoc* tests for energy expenditure, VO2 relative to the body mass, RER, blood lactate concentration, and MVC after exercise for knee extension and elbow flexion (Vincent, 2005). Statistical analyses were performed using SPSS software (version 15.0; SPSS, Tokyo, Japan).

## Results

**FIGURE 3:**
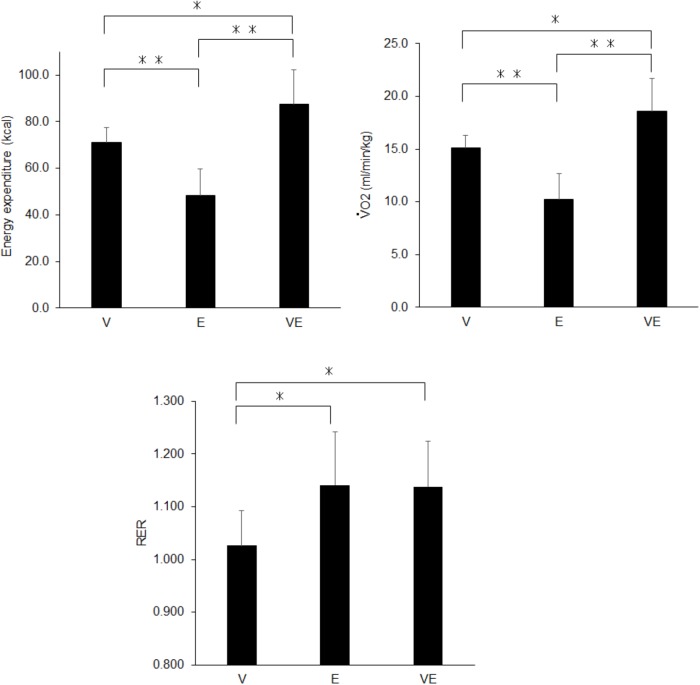
Energy expenditure (Left upper panel), $\dot{\text{V}}{\text{O}}_{2}$ relative to the body mass (Right upper panel), and the respiratory gas exchange ratio (RER) (Bottom panel) during voluntary aerobic exercise (V), whole-body neuromuscular electrical stimulation (E), and their combination (VE). ^∗^*p* < 0.05, ^∗∗^*p* < 0.01.

**FIGURE 4:**
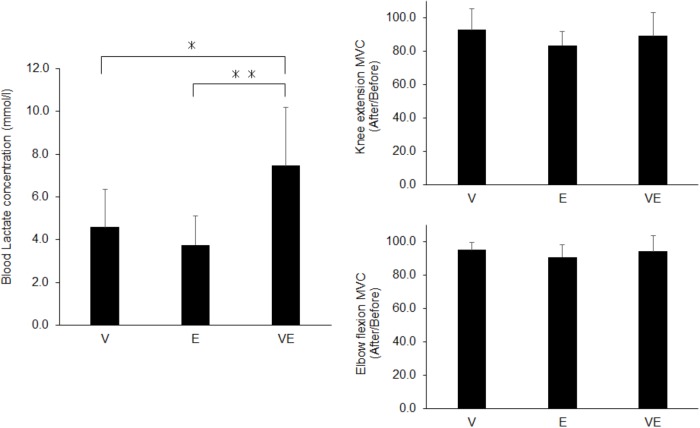
Blood lactate concentration (Left panel) and maximal voluntary contraction (MVC) after the exercises normalized by that before exercises (Right panels) for voluntary aerobic exercise (V), whole-body neuromuscular electrical stimulation (E), and their combination (VE). ^∗^*p* < 0.05, ^∗∗^*p* < 0.01.

There were significant effects of the exercise type on energy expenditure (*n* = 13, df = 2, *p* < 0.0001, *F* = 843.479, ES = 0.986), $\dot{\text{V}}{\text{O}}_{2}$ relative to the body mass (*n* = 13, df = 2, *p* < 0.0001, *F* = 881.063, ES = 0.987), RER (*n* = 13, df = 2, *p* < 0.0001, *F* = 3471.804, ES = 0.997), and blood lactate concentration after exercise (*n* = 13, df = 2, *p* < 0.0001, *F* = 142.849, ES = 0.923) (Figure 3, 4). Energy expenditure during VE was significantly higher than during V and E and that during V was significantly higher than during E (*n* = 13, *p* < 0.001, *F* = 39.687, *ES* = 0.878) (Figure 3). $\dot{\text{V}}{\text{O}}_{2}$ relative to the body mass during VE was significantly higher than during V and E, and that during V was significantly higher than during E (*n* = 13, *p* < 0.001 for VE vs. E and V and E, *p* < 0.001 for VE vs. V, F = 39.037, ES = 0.877) (Figure 3). RER during E and VE was significantly higher than during V (*n* = 13, *p* = 0.001, *F* = 11.73, ES = 0.681) (Figure 3). The blood lactate concentration after VE was significantly higher than after V and E (*n* = 13, *p* = 0.001, *F* = 13.873, ES = 0.716) (Figure 4). There were no significant differences among the exercises in MVC after exercise for knee extension and elbow flexion (*p* > 0.05) (Figure 4). The metabolic equivalents for V, E, and VE were 4.31 ± 0.35, 2.93 ± 0.71, and 5.31 ± 0.89 Mets, respectively. Statistical power in *post hoc* tests for energy expenditure, VO2 relative to the body mass, RER, blood lactate concentration, and MVC after exercise for knee extension and elbow flexion were 87.1 ± 12.4 (67.1-100.0).

$\dot{\text{V}}{\text{O}}_{2}$ relative to the body mass of VE exercise (18.6 ± 3.1 ml/min/kg) was significantly lower than the summation of $\dot{\text{V}}{\text{O}}_{2}$ relative to the body mass of V and E (21.8 ± 3.3 ml/min/kg) (*n* = 13, df = 12, *p* = 0.007).

## Discussion

We investigated metabolic responses and muscle fatigue during WB-NMES, voluntary exercise, and their combination. The main findings of the present study were 1) $\dot{\text{V}}{\text{O}}_{2}$ relative to the body mass during VE was greater than during V and E (*p* < 0.05) (Figure 3) and was lower than the summation of those during V and E, 2) RER during E and VE was higher than during V (*p* < 0.05) (Figure 3) and the blood lactate concentration after VE was markedly higher than after V and E (*p* < 0.05) (Figure 3, 4) there were no significant differences in MVC after the exercises (*p* > 0.05) (Figure 4). The results support the former part of hypothesis 1 that energy expenditure during the combination of voluntary exercise and WB-NMES is greater than during WB-NMES and voluntary exercise, but not the latter part of the hypothesis that energy expenditure during the combination of exercise and WB-NMES is greater than their summation. While hypothesis 2 was not supported, hypothesis 3 whereby the addition of WB-NMES to voluntary exercise enhances glucose metabolism was supported by the results of the present study.

Greater energy expenditure during the combination of voluntary exercise and WB-NMES (VE) compared with the separately performed voluntary exercise (V) and WB-NMES (E), as shown in the present study (Figure 3), is reasonable, since NMES can enhance energy expenditure by itself (Hamada et al., 2004a). We already demonstrated an increase in energy expenditure by the addition of NMES to leg muscles during aerobic pedaling exercise on a cycle ergometer (Watanabe et al., 2014). Another study also reported that the addition of WB-NMES leads to a 20% increase in energy expenditure during voluntary exercise (Kemmler et al., 2012). Since the increase in energy expenditure by the addition of WB-NMES was approximately 23% in the present study (Figure 3), VE was comparable to the combined program of voluntary exercise and WB-NMES used in a previous study by another research group (Kemmler et al., 2012). Thus, we suggest that the addition of WB-NMES increases energy expenditure during voluntary exercise. On the other hand, we found that this increase in energy expenditure due to the addition of WB-NMES could not be simply explained by the summation of energy expenditures from performing the two exercises. Our results showed that $\dot{\text{V}}{\text{O}}_{2}$ relative to the body mass during the combination of WB-NMES and voluntary exercise (VE) (18.6 ± 3.1 ml/min/kg) was significantly lower than those for WB-NMES (E) and voluntary exercise (V) that were separately performed (25.3 ± 3.3 ml/min/kg) (*p* < 0.05). We consider that wearing the suits with electrodes and the muscle contraction elicited by NMES may restrict the range of motion for voluntary exercise. While our study did not measure joint kinematics during the exercises, the range of motion during VE may be more restricted than that during V. This should be noted when the combination of WB-NMES and voluntary exercise is applied as an exercise tool.

The present study showed characteristic metabolic responses during the combination of WB-NMES and voluntary exercise compared with separately performed voluntary exercise and WB-NMES. RER during VE were significantly higher than V exercise (*p* < 0.05) (Figure 3). This finding is consistent with our previous study that NMES to leg muscles was applied during pedaling exercise (Watanabe et al., 2014). Also, RER during E, which did not involve any voluntary contractions has greater compared with that of V. Greater RER means an increase in the CO_2_ concentration in expired gas, which occurs with the enhancement of anaerobic energy metabolism. It is well-known that NMES can enhance anaerobic energy metabolism due to the recruitment of high-threshold motor units or muscle fibers associated with glucose metabolism (Hamada et al., 2004a,b; Gregory and Bickel, 2005; Jubeau et al., 2007; Bickel et al., 2011). Thus, our results suggest that WB-NMES can increase anaerobic energy metabolism irrespective of whether or not aerobic voluntary exercise is simultaneously applied.

The blood lactate concentration which is also indicator of the metabolic response, was significantly greater in VE than in V and E (*p* < 0.05) (Figure 4). This could also be due to NMES-induced recruitment of high-threshold motor units and muscle fibers (Hamada et al., 2004a,b; Gregory and Bickel, 2005; Jubeau et al., 2007; Bickel et al., 2011). On the other hand, there was a significant difference in the blood lactate concentration between VE and E (*p* < 0.05) (Figure 4), while RER was not significantly different between them (*p* > 0.05) (Figure 3). The blood lactate concentration is determined by both the production and consumption of lactate in metabolic systems. Lactate can be utilized as an energy source by skeletal muscles under high tissue-oxygen conditions, i.e., during voluntary exercise, while it cannot be consumed and accumulates in the blood under low tissue oxygen conditions, i.e., during anaerobic exercise (Brooks, 1986). A greater blood lactate concentration in VE compared with E may be explained by a decrease in lactate consumption during VE due to low tissue-oxygen conditions induced by the combination of voluntary exercise and WB- NMES. Also, it should be noted that the blood lactate concentration after VE was markedly increased in the present study, i.e., 7.5 ± 2.7 mmol/l (Figure 4). The metabolic cost during VE in this study was 5.31 ± 0.89 Mets, calculated from $\dot{\text{V}}{\text{O}}_{2}$, and this metabolic cost corresponds to that during brisk walking (Jette et al., 1990). Since blood lactate concentration of 7.5 mmol/l is observed during high-intensity exercise such as high-intensity interval training (Faude et al., 2009), the combination of voluntary exercise and WB-NMES can enhance the characteristic metabolic response, which cannot be explained by the relationship between the metabolic cost and blood lactate concentration during voluntary exercise.

We estimated energy expenditure, oxygen consumption, and metabolic characteristics by the method of spirometry. As shown in the result of blood lactate concentration, more than 7 mmol/l was observed in VE in the present study (Figure 4), indicating that a high anaerobic fraction during metabolism were recruited during this type of exercise. Under the exercise with anaerobic metabolism is enhanced, measurements of metabolic responses with the method of spirometry may underestimates the actual energy consumption, because anaerobic metabolism could be a delayed effect on the respiratory gases (Green and Dawson, 1993). Therefore, energy consumption may be underestimated in VE that recruited anaerobic metabolism in the present study. This would be limitation of this study and it should be noted that our results include this methodological issue in the calculation of metabolic responses and energy expenditure, in particular VE exercise. Significant lower oxygen consumption in VE comparing with the summation of V and E may be partly explained by the underestimation due to the methodology. In the future studies, we need to measure the metabolic responses after exercises to quantify the oxygen required to eliminate the “oxygen debt” for a better approximation of the actual energy consumption.

We assessed local neuromuscular fatigue using a comparison of MVC before and after the exercises. MVC decreased by approximately 10% after all exercises (Figure 4), and so all would induce muscle fatigue. However, there were no significant differences among the exercise types in MVC after the exercises for knee extensor and elbow flexor muscles (*p* > 0.05) (Figure 4). Considering that the blood lactate concentration was significantly higher in VE compared with V and E (*p* < 0.05) (Figure 4), a greater decrease in MVC after VE should be observed. This difference in results between local muscle fatigue assessed by MVC and blood lactate concentration could be mainly explained in two ways. First, while we applied NMES to nine muscle groups of the upper body, trunk, and lower extremities, MVC was measured for only two muscle groups. We thus estimated that differences in local neuromuscular fatigue among the exercises may occur in other muscle groups. Second, as stated above, both the production and consumption of lactate in metabolic systems contribute to the blood lactate concentration (Brooks, 1986). The greater blood lactate concentration in VE compared with V and E under similar local neuromuscular fatigue among the three exercises assessed by MVC may be explained by differences in lactate consumption between VE and V and E, as discussed above.

## Conclusion

The present study revealed that the found that combination of aerobic exercise and WB-NMES leads to greater energy expenditure, an increase in RER in expired gas, and a marked increase in the blood lactate concentration when comparing with those of separately performed aerobic exercise and WB-NMES. These results suggest that the combination of voluntary exercise and WB-NMES can enhance the metabolic response to a level equivalent to that of high-intensity exercise under the net physiological burden of low-middle-intensity exercise. This type of exercise would be useful for individuals who are unable to perform high-intensity exercise requiring anerobic metabolism or the recruitment of high-threshold motor units/muscle fibers such as type 2 diabetes mellitus patients and/or older adults.

## Data Availability

All datasets generated for this study are included in the manuscript and/or the supplementary files.

## Author Contributions

KW, SK, and TM planned the research. KW, SK, TY, and TI conducted the experiments. KW, TY, and TI analyzed the data. KW, SK, TY, TI, and TM discussed the results. KW wrote the manuscript. KW and TM edited and reviewed the manuscript.

## Conflict of Interest Statement

SK is employed by company MTG Co., Ltd. All other authors declare no competing interests.

## Abbreviations

MVC: maximal voluntary contraction
NMES: neuromuscular electrical stimulation
RER: respiratory gas exchange ratio
$\dot{\text{V}}{\text{O}}_{2}$: oxygen uptake
WB-NMES: whole body neuromuscular electrical stimulation
